# Large introns in relation to alternative splicing and gene evolution: a case study of *Drosophila bruno-3*

**DOI:** 10.1186/1471-2156-10-67

**Published:** 2009-10-19

**Authors:** Nikolai P Kandul, Mohamed AF Noor

**Affiliations:** 1 Biology Department, Duke University, PO Box 90338, FFSC 4244, Durham, NC 27708, USA; 2 California Institute of Technology, Biology, MC 156-29, 1200 E California Boulevard, Pasadena, CA 91125, USA

## Abstract

**Background:**

Alternative splicing (AS) of maturing mRNA can generate structurally and functionally distinct transcripts from the same gene. Recent bioinformatic analyses of available genome databases inferred a positive correlation between intron length and AS. To study the interplay between intron length and AS empirically and in more detail, we analyzed the diversity of alternatively spliced transcripts (ASTs) in the *Drosophila *RNA-binding *Bruno-3 *(*Bru-3*) gene. This gene was known to encode thirteen exons separated by introns of diverse sizes, ranging from 71 to 41,973 nucleotides in *D. melanogaster*. Although *Bru-3'*s structure is expected to be conducive to AS, only two ASTs of this gene were previously described.

**Results:**

Cloning of RT-PCR products of the entire ORF from four species representing three diverged *Drosophila *lineages provided an evolutionary perspective, high sensitivity, and long-range contiguity of splice choices currently unattainable by high-throughput methods. Consequently, we identified three new exons, a new exon fragment and thirty-three previously unknown ASTs of *Bru-3*. All exon-skipping events in the gene were mapped to the exons surrounded by introns of at least 800 nucleotides, whereas exons split by introns of less than 250 nucleotides were always spliced contiguously in mRNA. Cases of exon loss and creation during *Bru-3 *evolution in *Drosophila *were also localized within large introns. Notably, we identified a true *de novo *exon gain: exon 8 was created along the lineage of the *obscura *group from intronic sequence between cryptic splice sites conserved among all *Drosophila *species surveyed. Exon 8 was included in mature mRNA by the species representing all the major branches of the *obscura *group. To our knowledge, the origin of exon 8 is the first documented case of exonization of intronic sequence outside vertebrates.

**Conclusion:**

We found that large introns can promote AS via exon-skipping and exon turnover during evolution likely due to frequent errors in their removal from maturing mRNA. Large introns could be a reservoir of genetic diversity, because they have a greater number of mutable sites than short introns. Taken together, gene structure can constrain and/or promote gene evolution.

## Background

Alternative splicing (AS) and other post-transcriptional modifications of the same pre-mRNA generate structurally and functionally distinct transcripts from the same gene and thus could account for the origin of high protein diversity from the limited number of coding genes (e.g., [1-4]). The genome of *Drosophila melanogaster *[5] contains 15,182 annotated genes (Release 5.2 [6]) that encode 43,129 distinct transcripts (UniGene build #64 [7]). Therefore, on average, one *Drosophila *gene encodes 2.8 known or predicted transcripts. The ratio of genes to transcripts is even higher in humans, 1:9.5; 20,500 genomic genes [8] and 195,727 distinct transcripts (UniGene build #219 [7]).

The complex molecular structure of genes might be especially conducive to AS. In particular, exon number and intron length correlate with AS [e.g., [9-12]]. Genes with greater numbers of exons can generate more alternatively spliced transcripts (ASTs) just by possible combinations of a gene's exons [e.g., [10]]. One of the most extreme examples of *Drosophila *multi-exonic genes is *Dscam*. This gene potentially encodes 38,016 distinct axon guidance receptors via differential inclusion of its 95 exons [13]. Multiple recent bioinformatics analyses of genome databases inferred that exons surrounded by long introns (i.e., >250 bp) are skipped more often in gene transcripts than exons flanked by short introns [11,12,14,15]. In addition, recursive splicing of extremely large introns [16,17] may be prone to errors, which may lead to both exon-skipping and intron retention in transcripts. Therefore, the presence of many exons separated by long introns in a gene can promote, or at least associate with, the AS of a gene.

We studied the potential interplay between the intron length and AS using the *Drosophila Bruno-3 *(*Bru-3*). This gene has a genetic structure unusual for *Drosophila*. While the average *Drosophila *genes tend to have 4 exons and 487 bp-long introns [18], *Bru-3 *is encoded by 13 exons that spread along 129 kb of genomic sequence and its longest known mature mRNA is only 2.6 kb long. The lengths of the twelve introns in this gene are extremely diverse and range from 71 bp up to 41,973 bp (FlyBase.org, *D. melanogaster *release 5.13). Therefore, the inclusion frequency of exons surrounded by differently sized introns can be examined within this single gene. Only two alternatively spliced transcripts (ASTs) of *Bru-3 *- Bru-3-RA (2606 bp) and Bru-3-RB (2429 bp) - were identified and confirmed in *D. melanogaster *(UniGene Dm.13624). One additional alternative transcript can be inferred from 17 ESTs available at UniGene Database [7] that match the *Bru-3 *genomic sequence. However, this gene might have a greater diversity of ASTs than previously documented. By focusing our analysis to a single interesting gene, we sought to more thoroughly sample AST diversity directly and study the interplay between intron length and AS.

*Drosophila Bru-3 *belongs to a complex and diverse group of RNA binding proteins [e.g., [19]]. It encodes two RNA recognition motifs (RRMs), one at the N-terminus and one at the C-terminus of the gene, and a linker region between the RRMs [20,21]. *Bru-3 *was classified as the *Drosophila *orthologue of human and mouse *CUG-BP *(also called as *TNRC4*) and *Xenopus EDEN-BP *[i.e., Bluno-like proteins, [20,22]]. While it was shown that *Bru-3 *represses the translation of target genes by binding to the 3' UTR of their mRNA in *Drosophila*, [e.g., [20]], it may also regulate pre-mRNA AS and be involved in mRNA editing, as done by other members of this protein family [e.g., [19,23,24]]. The importance of these regulatory functions of *Bru-3 *is likely reflected in high sequence conservation at both coding and non-coding regions of this gene [but see also, [25]]. *Bru-3 *has the highest number of ultra-conserved elements per gene between *D. melanogaster *and *D. pseudoobscura*, separated 55 million years (MY) ago [26]. These elements comprise 11.2 kb [27], while the longest mature mRNA of *D. melanogaster *Bru-3 is only 2.6 kb (FlyBase.org, release 5.13).

Common computational methods rely on expression sequence tags [EST, [28]] to infer ASTs. As a result, inherent biases of EST recovery [for more detail refer to: [3,14,29,30]] lead to underestimation of the true number of alternatively spliced genes and especially the diversity of alternative transcripts of individual genes [e.g., [31,32]]. Direct bioinformatic identification of alternative splicing events, including cassette exons, alternative 5' and 3' splice sites, is still not totally accurate and comprehensive [31,32]. Application of microarrays for probing the diversity of alternative transcripts requires knowing the locations of exon-exon and exon-intron junctions [29,33]. In addition, hybridization-based methods tend to have low sensitivity and specificity [30,34,35]. Finally, the above methods rely on short sequences (i.e., perhaps 500-800 bp maximum) to infer particular exon combinations, and long-range contiguity of splice choices along a maturing mRNA cannot be extracted from these data. Therefore, detailed study of candidate genes using classic molecular methods, like RT-PCR of an entire transcript, is the method of choice when the entire diversity of alternative transcripts is investigated.

Here, the diversity of *Bru-3 *ASTs was analyzed via RT-PCR in four *Drosophila *species - *D. melanogaster, D. persimilis, D. pseudoobscura *and *D. virilis*. These species represent three distantly related *Drosophila *groups that were separated by 55 million years of independent evolution [26]. Thus the evolutionary conservation of ASTs of *Bru-3 *along such divergence would suggest their functional significance [e.g., [36]]. We also studied the molecular evolution of the translated part of *Bru-3*, including patterns of exon gains and losses, using the published genome sequences of twelve *Drosophila *species [37]. Roy et al. [15] found that long introns were enriched with newly created exons compared to short introns. Our data presented an exciting opportunity to analyze the patterns of exon gains and losses in regards to intron lengths. Finally, we tested the effect of splicing constraint (i.e., constitutive versus alternative splicing) on the evolution of homologous RRM domains in *Bru-3 *among twelve different species.

## Results

### Molecular structure and AS of *Bru-3 *in *Drosophila *species

#### The structure of *Bru-3*

**Figure 1:**
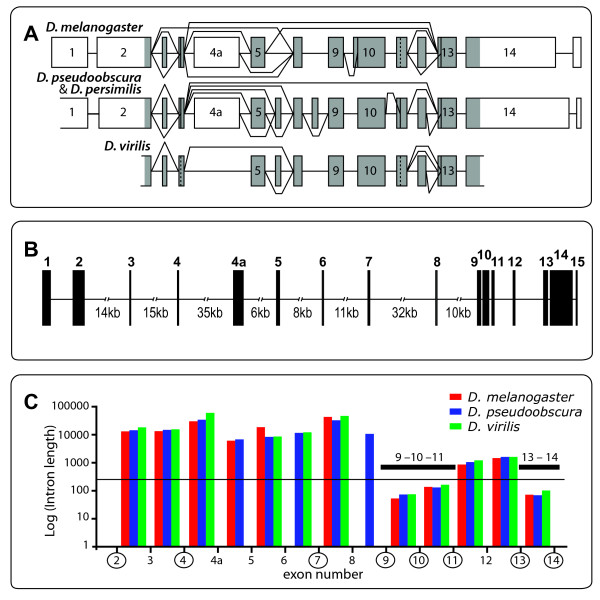
**Intron length and alternative splicing of *Bru-3 *in distantly related *Drosophila *species**. (*A*) Splice choices identified in *D. melanogaster, D. pseudoobscura, D. persimilis *and *D. virilis*. Only lengths of exons (boxes), but not introns (lines connecting exons), are drawn to scale. The ORF is shaded. The untranslated regions (UTRs) of *Bru-3 *were not analyzed in *D. virilis*. For simplicity, exon skipping and alternative acceptor sites, but not intron splicing *per se*, are depicted. While many splice choices in *Bru-3 *were found conserved between tested species, the gene underwent evolution at the exon level. Exon 8 were created *de nove *from intronic sequence in *D. pseudoobscura *and *D. persimilis *sibling species pair, and exon 6 lost coding potential in *D. melanogaster*. (*B*) The gene structure of *Bru-3 *in *D. pseudoobscura*. All exons and the introns that are shorter than 2 kb are drawn to scale. The lengths of the other introns are presented. (*C) *The intron length and splicing of adjacent exons in *Bru-3*. The column heights are proportional to the natural logarithm of intron lengths in *D. melanogaster, D. pseudoobscura *and *D. virilis*. The columns are aligned between corresponding exons. The circles enclose the number of exons that included into the majority of ASTs. The horizontal line depicts the suggested cut-off, 250 nucleotides, between short- and long- introns [12,54]. The shorter introns are likely spliced out through the intron definition mechanism whereas the longer introns relay on the exon definition mechanism for their splicing [12]. The intron definition mechanism of splicing was shown to be more precise and efficient than the exon definition mechanism [12]. We found that the exons adjacent to long-introns were frequently skipped in ASTs of *Bru-3*, whereas the exons connected by short-introns - exons 9, 10 and 11, and exons 13 and 14 (black bars) - were always spliced together.

The highest number of exons in *Bru-3 *was found in *D. pseudoobscura *and *D. persimilis*. A total of sixteen exons encode *Bru-3 *in these species (Figure 1A and Additional file 1). Of these, thirteen exons encode parts of the open reading frame. The remaining three exons either entirely form UTRs (i.e., exons 1 and 15) or are only occasionally retained in the mature mRNA and do not have a consistent, long ORF (i.e., 'nonsense' exon 4a in Figure 1A). Single PCR products were amplified from the 5'- and 3'-end UTRs of *Bru-3 *in *D. pseudoobscura *and *D. persimilis*, and thus the exons encoding the UTRs - exons 1, 2, 14 and 15 - were constitutively spliced in both species. Only single synonymous substitutions in the ORF and a few short insertions/deletions and substitutions in the 5'- and 3'-end UTRs distinguish both *D. pseudoobscura *isolates and *D. persimilis *MSH93. We identified a single nucleotide insertion in the published genomic sequence of *D. persimilis *at exon 10, which introduces two premature termination codons (PTCs) and thus was likely a sequencing or assembly mistake (scaffold 36 at position 405678, initial assembly; Flybase.org). The longest ORF of 1281 nucleotides was identified in *D. pseudoobscura*, transcript #1 in Table 1. Still, exon 3 was skipped in this transcript, and thus the longest potential ORF of *Bru-3 *in *D. pseudoobscura *and *D. persimilis *is 1323 nucleotides. The shortest ORF was identified in *D. persimilis *and *D. melanogaster*, and was only 414 nucleotides (transcript 33 in Table 1). Four translated exons were included in the ORF of this transcript, exons 2, 4, 13 and 14. Only two exons from the ORF of *Bru-3 *(13 and 14) were constitutively spliced and translated in all tested species (Table 1). The other exons of the ORF were skipped in at least one AST and thus were alternatively spliced exons *sensu stricto*.

**Table 1:** Diversity of alternatively spliced transcripts of *Bruno-3 *identified in four different species of *Drosophila*

		**Size of translated exon (bp)**														**Number of sampled clones**			
		
**AST**	**Size of ORF**	**2**	**3**	**4**	**4a**	**5**	**6**	**7**	**8**	**9**	**10**	**11**	**12**	**13**	**14**	***D. mel*. (19)**	***D. pse*. (51)**	***D. per*. (11)**	***D. vir*. (19)**
1	1281	60	-	49	-	135	-	80	57	144	274	99	78	177	128	N/A	1		N/A
2, A	1269	60	42	49	-	135	-	80	-	144	277	99	78	177	128	X			
3	1251	60	-	49	-	135	-	80	-	144	301	99	78	177	128	1	N/A	N/A	N/A
4*	1227/1224	60	-	49	-	135	-	80	-	144	277/274	99	78	177	128	2	4	2	4
5*	1206	60	-	49	-	135	-	80	-	144	274	99	78	159	128		3		3
6	1188	60	42	49	-	135	-	80	-	144	274	99	-	177	128	X	1	X	X
7	1173	60	-	49	-	135	-	80	-	144	301	99	-	177	128	2	N/A	N/A	N/A
8	1155	60	-	31	-	135	-	80	-	144	301	99	-	177	128	1	N/A	N/A	N/A
9*	1149/1146	60	-	49	-	135	-	80	-	144	277/274	99	-	177	128	2	8	1	2
10	1146+454	60	-	49	454	135	-	80	-	144	274	99	-	177	128		3		
11	1131	60	42	49	-	-	-	80	-	144	274	99	78	177	128		1		
12	1128	60	-	49	-	135	-	80	-	144	274	99	-	159	128		1		
13	1122	60	-	49	-	-	51	80	-	144	274	99	78	159	128	N/A	1		
14	1122	60	-	31	-	-	51	80	-	144	274	99	78	177	128	N/A	1		
15	1113	60	42	49	-	-	-	80	-	144	274	99	78	159	128		1		
16	1104	60	42	49	-	-	51	80	-	144	274	99	-	177	128			1	
17*, B	1092/1089	60	-	49	-	-	-	80	-	144	277/274	99	78	177	128	1	8	4	2
18	1089+452	60	-	49	452	-	-	80	-	144	274	99	78	177	128			1	
19*	1071	60	-	49	-	-	-	80	-	144	274	99	78	159	128		3		1
20	1071	60	-	31	-	-	-	80	-	144	274	99	78	177	128		1		
21	1062	60	-	49	-	-	51	80	-	144	274	99	-	177	128	N/A	1		
22	1062+454	60	-	49	454	-	51	80	-	144	274	99	-	177	128	N/A	1		
23	1059	60	-	49	-	-	-	80	-	144	274	69	78	177	128		1		
24	1053	60	42	49	-	-	-	80	-	144	274	99	-	177	128				1
25	1044	60	-	49	-	-	51	80	-	144	274	99	-	159	128		1		
26	1038	60	-	49	-	-	-	80	-	144	301	99	-	177	128	1	N/A	N/A	N/A
27	1026	60	-	31	-	-	51	80	-	144	274	99	-	159	128	N/A	1		
28*	1014/1011	60	-	49	-	-	-	80	-	144	277/274	99	-	177	128	2	7	1	5
29*	1014/1011 +454/452	60	-	49	454/452	-	-	80	-	144	277/274	99	-	177	128	1	1		
30*	996/993	60	-	49	-	-	-	80	-	144	277/274	99	-	159	128	1	1		1
31	965	60	-	-	-	-	-	80	-	144	277	99	-	177	128	1			
32	549	60	-	49	-	135	-	-	-	-	-	-		177	128	1			
33*	414	60	-	49	-	-	-	-	-	-	-	-		177	128	2		1	
34	378+454	60	-	31	454	-	-	-	-	-	-	-	-	159	128	1			
35	?					135	51	80								N/A	X	X	X

Alternatively spliced transcripts (AST) were analyzed in four different species of *Drosophila*: *D. pseudoobscura *(*pse*), *D. persimilis (per), D. melanogaster (mel) *and *D. virilis (vir)*. The total number of sequenced transcripts is shown in brackets for each species. Two known ASTs of *D. melanogaster *(FlyBase.org) labeled with letters - A and B - are also included in the table. *D. melanogaster *exon 10 was longer by 3 nucleotides than the same exon in the other species. Thus, two sizes separated by a slash are presented for the ASTs shared between both species. Although we have not sequenced the entire ASTs 2 and 35, the splice choices specific for these ASTs were sampled for species designated by "X".

* - AST was conserved between at least two distantly related *Drosophila *species.

#### Complex AS of *Bru-3*

Multiple ASTs of *Bru-3 *are expressed in adult flies of *D. melanogaster*, *D. pseudoobscura*, *D. persimilis *and *D. virilis*. We identified 22 distinct ASTs from the alignment of 49 sequenced clones of the entire ORF from *D. pseudoobscura *female and male flies [GenBank: GQ497346-GQ497396]. A smaller number of clones were sequenced for the other *Drosophila *species, and thus fewer distinct ASTs of *Bru-3 *were found for these species. 15 different ASTs were sampled for *D. melanogaster*, 8 ASTs for *D. virilis*, and 6 ASTs for *D. persimilis *(Additional file 2, GenBank: GQ497397-GQ497445). In addition, a few new exon combinations that represent new ASTs were inferred using primers bound to specific exons, and further increased the number of ASTs found in each *Drosophila *species (Additional file 2). Altogether, 35 distinct ASTs of *Bru-3 *have been identified across four *Drosophila *species (Table 1).

Multiple splicing events - exon skipping, alternative start codons, and alternative 3' splice sites (SS) - contributed to the diversity of transcripts of this gene. Although the diversity of ASTs of *Bru-3 *is likely underestimated for species other than *D. pseudoobscura*, the evolutionary conservation of individual splicing events and multiple ASTs along tens of MY of evolution is obvious (Table 1 and Figure 1A). Nine ASTs and two specific exon combinations - exons 3, 4, 5 and exons 5, 6, 7 - have been conserved in at least two distantly related *Drosophila *species, separated by 55 MY [transcripts with stars in Table 1, [26]]. For example, one distinct transcript that contained 'nonsense' exon 4a was identified in *D. melanogaster *and *D. pseudoobscura *(Table 1: transcript 29). The same two alternative 5' SS in exons 4 and 13 were used in *D. pseudoobscura *and *D. melanogaster*, and thus 18 bp-long truncations at the 5'-ends of these exons may be functionally significant in both species. The latter 5' SS is also used by *D. virilis*. While the alternative 5' SS in exon 10 appears to be an apomorphy of the *melanogaster *group (see below), the sequence at the alternative 5' SS in exon 11 is conserved among the twelve *Drosophila *species [37] and may be used as a SS in other *Drosophila *species (Figure 1A). Overall, many ASTs as well as specific splicing events of *Bru-3 *are conserved between distantly related *Drosophila *species.

#### A few mature mRNA included 'nonsense' exon 4a

Six clones of the entire ORF of *Bru-3 *from *D. pseudoobscura *and *D. persimilis *included an additional sequence, called here 'nonsense' exon 4a. These clones were derived from four independent cDNA pools of both *D. pseudoobscura *isolates and *D. persimilis *MSH93, and represent distinct ASTs (transcripts 10, 18, 22, and 29 in Table 1). The inclusion of exon 4a into transcripts introduced multiple PTCs and thus possibly rendered them non-functional for protein translation (aka 'nonsense'). The sequence of exon 4a is highly conserved between *D. pseudoobscura *and *D. persimilis*. Only two insertion/deletions of a single nucleotide and 5 substitutions out of 454 nucleotides distinguish both species. Exon 4a derives from the longest intron in *Bru-3 - *40,652 bp - between exons 4 and 5; 6,664 nucleotides upstream of exon 5 in *D. pseudoobscura *(r2.3, FlyBase.org). Interestingly, the sequences that appeared homologous to 'nonsense' exon 4a in *D. pseudoobscura *were also identified in two ASTs of *D. melanogaster *(transcripts 29 and 34 in Table 1). *D. melanogaster *exon 4a had the same length, introduced PTCs, and originated from the extremely long intron of *Bru-3 *- 36,042 bp - between exons 4 and 5; 5,918 nucleotides upstream of exon 5 (r5.13, FlyBase.org). However, while exon 4a originated from the homologous positions in *D. pseudoobscura *and *D. melanogaster *genomes, exon 4a was not conserved between two *Drosophila *species at the sequence level. In fact, both sequences appeared totally unrelated. Because exon 4a does not have the splice sites matching both U2- and U12-classes of spliceosomes [38] in both *Drosophila *species: once retained it cannot be spliced out individually from maturing mRNAs to re-establish their protein-encoding potential.

#### Alternative start codon of *Bru-3*

We inferred an alternative start codon for *Bru-3 *located in exon 7. Exons 2 and 7 were spliced to each other in *D. melanogaster *transcript 31 (Table 1). Although skipping exon 4 offset the reading frame starting from exon 2, the alternative start codon located at the 3'-end of exon 7 could be used in transcript 31. The resulted polypeptide chain would skip the entire N-terminal RRM, but it would include the other functionally important domains of *Bru-3*, like the linker region and C-terminal RRM [20]. Therefore, transcript 31 may be a valid, albeit rare, transcript encoding a functional protein in *D. melanogaster*.

#### Exon 6 can be spliced in the middle of the N-terminal RRM

Each *Bru-3 *RRM is formed by two exons. Exons 5 and 7 together make the N-terminal RRM. We identified exon 6 in *D. pseudoobscura *that was located between these functionally linked exons in the genomic sequence and was spliced together with exon 7 (Table 1). However, none of the sequenced clones of *Bru-3 *had exon 6 retained between exons 5 and 7. To test whether the constraints acting on the functional domain would prevent the production of *Bru-3 *transcripts with exon 6 disrupting the RRM, we screened cDNA pools from *D. pseudoobscura*, and *D. persimilis *for transcripts with consecutive exons 5, 6 and 7. Amplicons with exons 5, 6 and 7, as well as exons 5 and 6 spliced together were found in both species (Additional file 3). Because exon 6 does not have a 3' SS, once retained between exon 5 and 7, it cannot not be spliced out by itself.

#### Intron size and AS in *Bru-3*

In the translated segment of *Bru-3*, exons separated by large introns were skipped more often than exons divided by small introns, less than 250 nucleotides. Furthermore, the consecutive exons that were always spliced together in ASTs were divided by three shortest introns in the gene - all less that 160 bp (Figure 1B, C) in the tested *Drosophila *species. The two shortest introns - introns 9 and 10 - divide exons 9, 10 and 11. These exons were always spliced together: if any of these three exons was included in the transcript the others were also included (Table 1). The third shortest intron - intron 13 - divides exons 13 and 14 (Figure 1B). These exons were included in every ASTs of *Bru-3 *identified in four *Drosophila *species (Table 1). The next shortest intron separating the translated exons in *Bru-3 *- intron 11 - was already unusually large for Drosophila (Figure 35 in [18]): 847 bp in *D. melanogaster*, and 1024 bp in *D. pseudoobscura*. Less than 9% of all *D. melanogaster *and *D. pseudoobscura *introns have the same or longer sizes, and 86 and 69 nucleotides are the median intron sizes is both species, respectively. Intron 11 divides exon 12 from the group of contiguously spliced exons 9, 10 and 11 (Figure 1B). Perhaps not surprisingly, exon 12 was skipped in more that half of the transcripts that included exons 9, 10 and 11 (Table 1). The 5'-end part of *Bru-3 *ORF - exons from 2 through 9 - has larger introns that the 3'-end part of ORF, and overall the higher number of AS events were identified in the 5'-end part of the ORF (Table 1). Nevertheless, exons 4 and 7, each located adjacent to large introns, were faithfully included into almost all surveyed transcripts, precluding statistically significance in a simple association between intron size and exon skipping.

### Evolution of *Bru-3 *in Drosophila

#### Exon turnover during Bru-3 evolution

**Figure 2:**
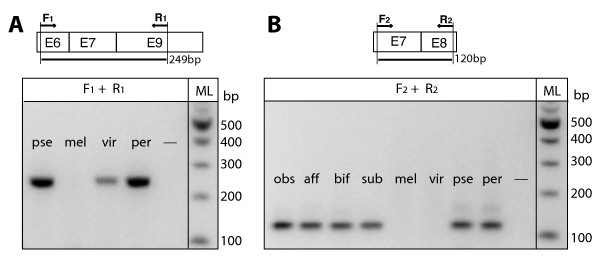
**cDNA screens for specific splice choices of *Bru-3***. In the diagrams, arrows indicate the position and direction of primers in regard to the exons (e.g., E6, E7, E8 and E9) used for PCR. The expected sizes of PCR products (in nucleotides) are presented below the diagram. In the gel images, fly lines are indicated for each track. The long dash marks the track of a negative control (no template). Sizes of DNA ladder (ML) are shown to the right of the gel image. (*A*) Screening cDNA pools for the splice variant containing exon 6. The exon was included in mature mRNAs of *D. pseudoobscura *(pse), *D. persimilis *(per) and *D. virilis *(vir), whereas it was absent in *D. melanogaster *(mel) cDNA pool. (*B) *Screening cDNA pools for the splice variant containing exon 8. Although exon 8 was included in mature mRNAs of all tested species of the *obscura *group - *D. obscura *(obs), *D. affinis *(aff), *D. bifasciata *(bif), *D. subobscura *(sub) - it was not found in cDNA pools of the species outside of the *obscura *group, in *D. melanogaster *(mel) and *D. virilis *(vir).

**Figure 3:**
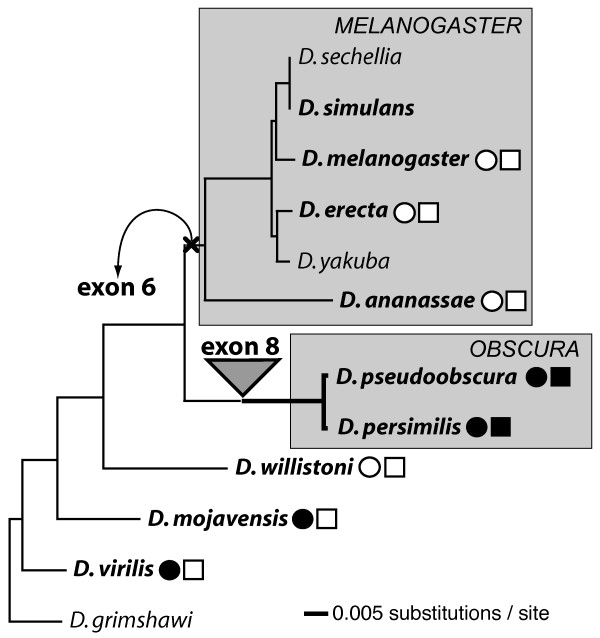
**Phylogenetic positions of exon gain and loss in *Bru-3***. The coding sequences of *Bru-3 *were inferred for 8 *Drosophila *species from their genomic sequenced available at FlyBase.org [37]. A distance tree was calculated in PAUP 4.0b10 [99] from the coding parts of eleven exons of *Bru-3 *(exons 2-5, 7, 9-14) shared by twelve *Drosophila *species. Applying nucleotide BLASTing [90], we found that exon 6 was lost along the branch leading to the *melanogaster *group (the top shaded box), whereas exon 8 was *de novo *created in the *obscura *group (the bottom shaded box). We verified both events for *Drosophila *species in bold by amplifying and aligning the orthologous genomic regions that include exons 6 and 8 (supplementary files S1 and S2). In addition cDNA pools from the species in bold, except for *D. simulans*, were screened for inclusion of conserved exon 6 and 8 in mature mRNA (fig. 2). The status of exon 6 indicated with a circle; a black circle, the exon was identified in cDNA pool; a white circle, the exon was absent. A square was used to indicate the status of exon 8.

Two previously unknown exons (6 and 8) of *Bru-3 *were identified in *D. pseudoobscura*. These exons encode short polypeptide chains in *Bru-3 *protein - 17 and 19 amino acids, respectively. No previously sampled ASTs from *Drosophila *species other than *D. pseudoobscura *included either of these exons (Table 1). However, we confirmed via RT-PCR that both exons are included in mature mRNA of *D. persimilis*, and exon 6 is included in mature mRNA of *D. virilis *(Figure 2A, B and Additional file 2). The location of each exon in the twelve published genomes of *Drosophila *species is indicated in Additional file 1. We have not found sequences homologous to exon 6 in species of the *melanogaster *group whose genomes were sequenced [37]. Likewise, the sequences homologous to exon 8 could not be found in *Drosophila *species other than *D. pseudoobscura *and *D. persimilis*. When we mapped the presence of exons 6 and 8 onto the phylogeny of twelve *Drosophila *species, we found that exon 6 was lost along the branch leading to the *melanogaster *group, while exon 7 appeared to be an apomorphy of the *obscura *group (Figure 3).

The proposed cases of exon loss and gain are not simple mistakes of genome assembly. We sequenced the genomic regions homologous by their positions to the regions that encompass exons 6 and 8 in *D. pseudoobscura *for *D. simulans, D. melanogaster, D. erecta, D. ananassae, D. pseudoobscura, D. persimilis, D. mojavensis *and *D. virilis*, and compared to their genomic counterparts. We have not found any mistakes of genome assembly that could explain the exon loss and gain in *Bru-3*, and thus the exon turnover is genuine. Screening of cDNA pools from *D. melanogaster, D. erecta, D. ananassae, D. pseudoobscura, D. willistoni*, *D. mojavensis*, and *D. virilis *for the presence of exons 6 and 8 further supported our conclusion. Exon 6 was identified in cDNA pools from *D. pseudoobscura, D. mojavensis*, and *D. virilis*, whereas exon 8 was found only in *D. pseudoobscura *cDNA pool (Figure 2A, B).

To investigate focal changes that could lead to the loss and gain of respective exons, we aligned the sequenced fragments encompassing exons 6 and 8 in *D. simulans, D. melanogaster, D. erecta, D. ananassae, D. pseudoobscura, D. persimilis, D. willistoni, D. mojavensis *and *D. virilis *(Additional files 4 and 5). Sequences clearly homologous to exon 6 were found in *D. willistoni, D. mojavensis*, and *D. virilis*. These sequences were conserved at both the amino acid and nucleotide levels. In contrast, although the tested species from the *melanogaster *group had the conserved 3' SS of the intron upstream of exon 6, the sequences homologous by position to exon 6 were not conserved in these species (Additional file 4). The region corresponding to exon 6 had an in-frame PTC in *D. melanogaster, D. simulans *and *D. ananassae*. In addition, *D. ananassae *had an insertion in this region. Therefore, the local deletions/insertions and/or substitutions at the 5' SS flaking exon 6 likely led to the loss of coding potential and subsequent decay of exon 6 in the *melanogaster *lineage (Figure 3). Higher sequence conservation was detected in the genomic fragments encompassing exon 8. The location of two 30 nucleotide-long ultraconserved regions near 5' SS facilitated the alignment of these fragments. Although all tested *Drosophila *species shared both 3' and 5' SSs flanking exon 8, the region corresponding to exon 8 in *D. pseudoobscura *and *D. persimilis *was not conserved at either amino acid or nucleotide levels in the other tested species (Additional file 5). We also found that *D. simulans*, *D. melanogaster*, *D. ananassae*, *D. mojavensis *and *D. virilis *all had at least one in-frame PTC, while *D. willistoni *had a 47 nucleotides-long insertion at the region corresponding to exon 8. Therefore, this region does not encode a polypeptide chain in any tested species except *D. pseudoobscura *and *D. persimilis*, and the ORF of exon 8 was likely created *in situ *from intron sequence in the *obscura *group.

#### Exon 8 originated early in the evolution of the *obscura *group

To study the origin of exon 8 in further detail, the genomic fragments encompassing exon 8 were sequenced for the species representing all major lineages in the *obscura *group [e.g., [39,40]]. All tested species from this group - *D. pseudoobscura bogotana*, *D. miranda*, *D. obscura, D. bifasciata, D. subobscura *and *D. affinis *- possessed the conserved exon 8. The last four of the above species each had one non-synonymous substitution in the 3'-end of exon 8 and a few substitutions at 3' and 5' SS around this exon (Additional file 6). To test whether these substitutions at the 3' and 5' SS could affect the inclusion of exon 8 into *Bru-3 *mRNA, we screened cDNA pools of *D. pseudoobscura *and *D. persimilis*, *D. obscura, D. bifasciata, D. subobscura *and *D. affinis *for its presence. Exon 8 was identified in the RNA pools of males and females from each tested *obscura *species, but not in *D. melanogaster *and *D. virilis *(Figure 2B). Therefore, exon 8 originated early in the lineage of the *obscura *group before the major branches [39,40] within this group appeared.

#### New 3' alternative splice site in exon 10

The longer version of exon 10 was identified in four ASTs of *D. melanogaster *(Table 1: transcripts 3, 7, 8 and 26). This version of exon 10 was 24 nucleotides longer that the previously known size of this exon in *D. melanogaster *(i.e., 277 nucleotides, FlyBase.org: r5.13). We analyzed the origin of the alternative 3' SS of exon 10 using the published genomes of *Drosophila *species [37]. While all twelve *Drosophila *species share the common 3' SS upstream of exon 10, an additional 3' SS was created along the lineage leading to the *melanogaster *group (Figure 3). The splicing at this new 3' SS would add 30 bp to *D. erecta *exon 10, 18 bp to *D. ananassae *exon 10, and 24 bp to exon 10 in the other four species in the *melanogaster *group (Additional file 7). While no potential 3' SS could be inferred in the homologous intron for *D. grimshawi, D, mojavensis, D. virilis*, and *D. willistoni*, three such sites were identified upstream of exon 10 in *D. pseudoobscura *and *D. persimilis*. However, we doubt that these additional 3' SS are active in both species. The minimum intron length required for successful splicing is around 45 nucleotides in *Drosophila *species [e.g., [41,42]], whereas these 3' SS upstream of exon 10 were only 15, 21, and 27 nucleotides apart from the 5' SS downstream of exon 9, and exons 9 and 10 were always spliced together (Tables 1). Therefore, the alternative 3' SS in exon 10 is a true apomorphy of the *melanogaster *group.

#### Splicing constraints affect the rate of sequence evolution

Splicing constraints (i.e., alternative or constitutive splicing) affect the evolutionary potential of a gene region. Constitutively spliced regions are expected to experience stronger functional constraints than alternatively spliced regions of the same gene [e.g., [10,43]]. *Bru-3 *allows the comparison of substitution patterns between two homologous domains present in the same gene that experience different splicing constraints: AS RRM versus CS RRM. Because these RRM were paralogous domains and thus potentially shared the common ancestry, this comparison controlled better for the variables that could bias the analysis. The AS RRM had more differences (i.e., accelerated evolution) in comparison to the CS RRM (i.e., AS RRM: 192 bp, 19.8% sites with substitution, across species = 0.0744 ± 0.0095, K_S _= 0.4034 and K_A _= 0.0340; CS RRM: 219 bp, 13.7% sites with substitution, across species = 0.0489 ± 0.0067, K_S _= 0.2047 and K_A _= 0.0), and this difference was marginally significant (G^2 ^= 2.75, p = 0.097).

## Discussion

Recent bioinformatic analyses of genome databases pointed to a correlation between intron length and alternative splicing [AS, e.g., [11,14,15]]. We studied in-depth the interplay between intron length and AS within the *Drosophila Bruno-3 *(*Bru-3*) gene. The molecular structure of this gene, multiple exons separated by large introns, was expected to be highly conducive to AS. Further, multiple *Bru-3 *exons are split by introns of remarkably diverse lengths (Figure 1B, Additional file 8), and thus presented a representative sample of intron sizes. Although only a single gene, extensive sampling of the diversity of *Bru-3 *ASTs across distantly related *Drosophila *species provided a case-study for several fundamental questions dealing with the interaction between gene structure, AS and gene evolution.

The sensitivity and specificity of our analysis of ASTs was accomplished by reverse transcription coupled with subsequent PCR and cloning. We identified 35 distinct ASTs of *Bru-3 *transcribed in *Drosophila *adult flies (Table 1), 33 of these ASTs were previously unknown. Notably, only seventeen ESTs of the 544,789 total *D. melanogaster *ESTs map to *Bru-3*, whereas for example, 403 ESTs map to *myosin light chain 2 *(UniGene build #59). It is well known that genes poorly represented in EST collections are expressed at a low level. However, such genes are more likely to be regulatory in function and undergo AS [e.g., [3]], like *Bru-3*. Cloning and sequencing of the complete ORF allowed the analysis of long-range contiguity of splice choices along the entire translated region of *Bru-3*. The contiguity of splice choices along a mature mRNA could not be inferred from either EST collections or by currently popular high-throughput methods; including classic microarrays, tiling arrays [e.g., [34,44,45]] and pyrosequencing of entire transcriptomes [e.g., [30]]; because frequencies of usage of individual splice choices and/or exons along mRNAs cannot be easily concatenated into distinct splicing variants. Below, we elaborate on several specific findings from our study.

### Conservation of alternative splice variants

High conservation of ASTs of a gene implies their functional significance [e.g., [36]]. We screened for *Bru-3 *ASTs in four *Drosophila *species. These species represented three distantly related species groups - *melanogaster*, *obscura*, and *virilis *- that were separated at least 55 million years (MY) ago [26]. *D. pseudoobscura *and *D. persimilis *are closely related species, which diverged less than 1 MY ago [26], and frequently share long haplotypes. Complex alternative splicing of *Bru-3 *was found in each tested species, and many individual ASTs and splicing events remained conserved along 55 MY of independent evolution (Table 1 and Figure 1A). For example, the short transcript of *Bru-3 *that included only 4 exons out of 13 translated exons (identified in *D. melanogaster *and *D. persimilis*) can be a functional transcript. Likewise, 18 nucleotide-long truncations in exons 4 and 13 may reflect either a function of the ASTs that includes these truncated exons or a common error-generating mechanism in *D. melanogaster *and *D. pseudoobscura*. The great diversity of conserved ASTs of *Bru-3 *uncovered in our study implied that this gene has a complex regulatory potential with many target genes. This spectrum of potentially distinct functions of *Bru-3 *is performed via ASTs of the same gene, not via gene duplications [46]. Therefore, many distinct transcripts need to be considered to study the function(s) of *Bru-3 *in *Drosophila*.

### Alternative splicing and accelerated evolution

Alternatively spliced exons (ASEs) are expected to undergo faster divergence than constitutively spliced exons (CSEs) at non-synonymous sites, because they are not included in every transcript of the gene and thus experience weaker functional constraints at the protein level [e.g., [43,47,48]]. The relaxation of functional constraints in ASEs can indirectly accelerate their evolution and permit additional diversification. Thus ASEs tend to have a higher frequency of non-synonymous substitutions than CSEs [e.g., [43,47-49]]. However, higher sequence conservation was previously found at synonymous sites in ASEs [e.g., [48,50]] and the introns flanking ASEs [e.g., [49,51]] than those in CSEs. To explain this counter-intuitive inference, Xing and Lee [43,48] suggested that ASEs experienced the stronger "RNA-level selection pressure" than CSEs, and thus ASEs had stronger conservation at the synonymous sites than CSEs. Indeed, AS requires the involvement of more regulatory elements than constitutive splicing [51,52]. However, it is unclear why "RNA-level selection pressure" would affect non-synonymous sites differently from synonymous sites. At the same time, ASEs tend to have smaller sizes than CSEs [e.g., [48,49,53]], and thus on average, splicing regulatory elements can occupy the higher proportion of the sequence in ASEs than those in CSEs. This bias was not always accounted for in bioinformatic analysis of exon collections [but see [49]].

The two paralogous RRMs in *Bru-3 *provided a system to test the effect of splicing constraints on the evolution, since their common ancestry supported the strongest control for potential biases. The C-terminal RRM was a CS domain, while the N-terminal RRM was an AS domain (Table 1). To account for the potential effect of exon size differences, we compared only the sequences of the RRM domains (AS RRM: 192 bp, CS RRM: 219 bp). The AS RRM experienced accelerated evolution compared with the CS RRM at both synonymous and non-synonymous sites (i.e., K_S _and K_A_). Therefore, after accounting for the different biases, we observed a trend indicating that the AS RRM experienced weaker purifying selection pressure at both synonymous and non-synonymous sites than the CS RRM in *Bru-3*, and thus the AS of the N-terminal RRMs could facilitate the evolution of an additional secondary specialization of this domain.

### Intron length can promote alternative splicing

The positive correlation between exon skipping and increasing lengths of the flanking introns [11,14] has been linked to the switch from the intron definition mechanism of splicing to the exon definition mechanism [12]. The intron definition mechanism operates effectively across short introns, whereas the exon definition becomes dominant when flanking introns are longer that 200-250 nucleotides [12], and thus splice sites are recognized across exons [42,54]. These mechanisms offer distinct predictions of splicing errors. Failures of the exon definition mechanism lead to exon skipping, while mistakes of splicing through the intron definition mechanism result in intron retentions [e.g., [11,54]]. However, splicing errors of extremely large introns via recursive splicing may also result in intron retentions. Fox-Walsh et al. [12] showed that the intron definition mechanism was more efficient at recognition of weak splice sites than the exon definition mechanism. The results of our analysis of AS in *Bru-3 *support these conclusions. The majority of exon skipping events mapped to the region of *Bru-3 *that included exons separated by long introns (Figure 1). Exons 2 through 9 in twelve *Drosophila *species [37] were separated by introns of at least 8,000 nucleotides (Additional file 8). In contrast, the translated exons that were always spliced contiguously in the four tested *Drosophila *species - exons 9, 10 and 11, and exons 13 and 14 - were separated by the smallest introns in the gene, each less than 200 nucleotides (Figure 1C). Therefore, the splicing of these exons was expected to proceed through the intron definition mechanism and thus be more efficient than that of other exons in the ORF of *Bru-3*. In the gene region between exons 9 and 14, only exon 12 was flanked by large introns (at least 847 nucleotides), and perhaps not coincidentally, exon 12 was skipped in more than a half of ASTs (Table 1). However, this trend was not perfect: exons 4 and 7 were included in almost all transcripts surveyed despite adjacency to large introns. Nonetheless, intron length can be important causal factor orchestrating the inclusion level of exons.

Intron length can also indirectly promote AS in general, because longer introns can support a finer level of AS regulation than the short introns. However, the alternative 3' SSs were identified equally frequently in both small and long introns of *Bru-3*: introns 9 and 10 versus introns 3 and 12, respectively (Figure 1A). Extremely large introns influence gene expression via their sheer size. For example, transcription of intron 7 in *Bru-3 *requires approximately 40 min [assuming 1 kb per min, [55,56]], and thus large introns can substantially delay mRNA maturation. Regulatory genes may use the interplay between transcript elongation rate and splicing kinetics to facilitate the regulation of AS [17,56-58]. In addition, long introns can contain many regulatory elements that are necessary to control AS at the tissue and temporal level [59,60]. Many regulatory genes known to have ASTs contain extremely large introns, including *muscleblind*, *Ultrabithorax*, *fruitless*, *kuzbanian*, and *pumilio *[UniGene and [17]].

### Intron length and the origin of coding diversity

The preferential gains of new exons within long introns can also explain the connection between exon-skipping and long introns. AS is strongly associated with recent exon origin [e.g., [33,43,47,61-64]] and the rate of exon gains is inversely related to exon inclusion level [e.g., [15,63,64]]. In other words, recently arisen exons tend to be included only in a few ASTs of a gene (i.e., be minor-form ASEs). Using phylogenetic analysis of 17 vertebrate genomes, Roy et al. [15] found that newly originated exons were more enriched within longer introns compared with short introns. Therefore, longer introns may have slightly higher probability of exon gains than shorter introns due to the higher number of mutable sites [15].

We identified one new putatively functional exon that was created from an intron sequence *de novo*, exon 8 in *D. pseudoobscura*. Although this exon is flanked by the 5' and 3' SSs which are conserved among *Drosophila *species separated by 60 MY of independent evolution [26], only the species of the *obscura *group have a true ORF between these SSs (Figure 2). The tested *Drosophila *species outside the *obscura *group all have a non-translated sequence at the homologous location to exon 8 (Additional file 5), in the largest intron of *Bru-3 - *41,973 bp in *D. melanogaster *and 45,859 bp in *D. virilis *(Figure 1C). As expected for a newly originated exon, exon 8 is a minor-form ASE. Only one sequenced clone of ORF of *Bru-3 *included exon 8 (Table 1), albeit subsequent screening found that this exon was included in mature mRNAs in both males and females of *D. pseudoobscura *and *D. persimilis*, as well as in the other four tested species from the *obscura *group (Figure 2B). Because all tested *Drosophila *species share some level of conservation between these SSs, and exon 8 is only 57 bp long, the sequence change that capacitated exon 8 were highly focal. Duplication-translocation of existing exons [e.g., [65,66]], retrotransposition [e.g., [67]] or exaptation of repetitive elements [e.g., *Alu *element, [62,68]] cannot explain the origin of exon 8. The functional ORF of exon 8 was created *de novo *from intronic sequence between the existing SSs that might previously function in recursive splicing of a huge intron [16,17]. The origin of exon 8 is especially remarkable, since all well-studied cases of *de novo *exon creation from intronic sequence are known from bioinformatic analyses of vertebrate genomes [e.g., [63,64,69]]. We are familiar with only one study in which five novel genes were inferred to originate from non-coding sequences in *D. melanogaster *[70]. However, unlike *Bru-3 *exon 8, four of these five genes had paralogous copies in the genome and thus their origins were associated with duplication-translocation.

The pre-existing location of sequences forming both SSs around exon 8 could play a critical role in the creation of this exon. In contrast, the deletion of one SS could instantly make the existing exon invisible to splicing machinery and thus would result in complete exon loss from mRNA and protein. The removal of translational constraint makes an exon sequence prone to mutational decay, akin to a pseudogene. We found one example of such 'turning off' of an existent exon in *Bru-3*. The deletion of the 3' SS flanking exon 6 along the lineage to the *melanogaster *group abolished its expression in this group, though this exon was included in mature mRNAs in *D. pseudoobscura, D. persimilis, D. virilis *and *D. mojavensis *and appeared functional in the other analyzed *Drosophila *species (Figures 2A and 3). Notably, the focal deletion of the 3' SS of exon 6 also took place between two large introns 5 and 6, which are 8463 bp and 1188 pb, respectively in *D. pseudoobscura*. Thus, this mutation also happened inside a large intron and led to the disappearance of exon 6 in the *melanogaster *group.

If large introns are conducive for exon gain and loss (i.e., exon turnover), we also expect to see more splicing mistakes in large introns compared to short introns. We identified one 'nonsense' exon 4a that originated from the largest intron of *Bru-3*. This exon was included into multiple ASTs in *D. pseudoobscura, D. persimilis *and *D. melanogaster*, and introduced multiple PTCs to mRNA (Table 1). Exon 4a is surrounded by conserved SSs in a proper orientation. The 3' SS located upstream of exon 4a can be a strong SS since it has a nearly 50 nucleotide-long pyrimidine stretch. This exon does not share major common characteristics of internal ASEs. First, its size is unusually large for internal ASEs in *Drosophila*. The average exon length in *D. melanogaster *is 150 nucleotides and fewer than 5% of exons have larger sizes than 454 nucleotides Figure 35 in [18]. Second, the total number of nucleotides in exon 4a is not divisible by three unlike in the majority of ASEs, especially in minor-form ASEs [e.g., [52,53,71]]. Both of these predicted features of ASEs result from the selection pressure to preserve an ORF (e.g., exons 3, 5, 6, 8 and 12 in Table 1). In addition, the sequence of exon 4a looks completely unrelated between *D. pseudoobscura *and *D. melanogaster*, while both species share the location of the SSs flanking this exon. These SSs are conserved between distant species because they likely function in recursive splicing of a 36 kb-long intron [17]. We think that the neighboring location of both SSs may bias toward the erroneous inclusion of the encompassed intronic sequence (i.e., 'nonsense' exon 4a) into *Bru-3 *mRNA.

Large introns have more opportinities to mutate than short introns due to the higher number of potentially mutable sites. In addition, the preferential location of large introns in genome areas with low recombination [72,73] can further increase the spread of new mutations within them compared to short introns. We know from our work on recombination variation in *D. pseudoobscura *that *Bru-3 *resides in an extremely cold recombination spot [74]. Notably, the deletion-biased mutation ratio (relative to insertions) known for *Drosophila *[75,76], which is expected to be especially strong in the areas of low recombination [77,78], has not led to the shortening of large introns in *Bru-3 *and other genes. At the same time, the presence of large introns in genes likely increases the energetic cost of their transcription, and thus highly expressed genes in humans, *Caenorhabditis *and *Drosophila *tend to have small introns [e.g., [60,79,80]]. Therefore, large introns either have some selective advantage or they can be more easily tolerated in some genes. Comeron and Kreitman [73] suggested that the large introns could decrease crossover interference between exons in the areas with low recombination [but see also, [72,81]], and thus they could be advantageous. Our data suggest that large introns can have yet another beneficial function. Large introns can contribute to the expansion of transcript diversity encoded by a single gene via both AS and *de novo *creation [82] or deletion of exons. Failures of proper splicing of large introns generate both exon skipping and intron retentions (i.e., via recursive splicing) in transcripts, which are screened out by nonsense-mediated mRNA decay (NMD) pathway [e.g., [83-85]]. The tolerance of suboptimal splicing efficiency and accuracy via NMD in turn may allow natural selection to 'sculpt' retained intronic sequences, like exon 4a in *Bru-3*, into a new translated exons, like exon 8 in *Bru-3*. Many species-specific ASEs in mammals were likely created *de novo *from purely intronic sequence [e.g., [15,63,64,86]] potentially by the similar mechanism.

## Conclusion

*Drosophila Bru-3 *has many exons that are separated by introns of extremely diverse lengths. The in-detail analysis of the ASTs of *Bru-3 *has inferred much greater diversity of ASTs that has been previously unknown. Our analysis of inclusion frequency of *Bru-3 *exons supported a positive correlation between exon skipping and surrounding intron length. The majority of cassette exons were located in the gene region with the longest introns, between exons 2 and 9. Conversely, the exons separated by short introns, i.e., less than 250 nucleotides, were always found splicing consecutively. In addition, cases of evolutionary gains and losses of exons were also mapped to long introns. Notably, we established that exon 8 was created *in situ *from intronic sequence positioned between cryptic SSs, which conserved among twelve *Drosophila *species. The presented findings support the role of gene structure in promoting and/or constraining gene's AS. However, a correlation between the intron length and AS does not imply the causality. Both gene structure and AS can be shaped by the interplay of selection, recombination and gene-gene interaction. Further study will determine whether the AS of *Bru-3 *is a general or special case.

## Methods

### *Drosophila *lines and their rearing

Two isolates of *D. pseudoobscura *- Mather 17 and Flagstaff 1993 [described in [87,88]], *D. melanogaster *(Oregon-R), *D. virilis *(San Diego Drosophila Stock Center accession [SDDSC] 15010-1051.87) and *D. persimilis *Mount St. Helena 1993 (MSH93) were used for analysis of the AST diversity of *Bru-3*. To study exon gains and losses during *Bru-3 *evolution in *Drosophila*, we examined additional lines - *D. simulans *(SDDSC 14021-0251.195), *D. erecta *(SDDSC 14021-0224.01), *D. ananassae *(SDDSC 14024-0371.13), *D. mojavensis *(SDDSC 15081-1352-26), *D. willistoni *(SDDSC 14030-0811.24), and species from the *obscura *group: *D. pseudoobscura bogotana *El Recreo line [89], *D. miranda *(SDDSC 14011-0101.08), *D. affinis *(SDDSC 14012-0141.04), *D. obscura *(SDDSC 14011-0151.01), *D. subobscura *(SDDSC 14011-0131.09), and *D. bifasciata *(SDDSC 14012-0181.02). All flies were maintained under a constant regime of temperature (20°C) and humidity (80%) in 12-h dark-light cycle. A standard mixture of agar, dextrose and yeast was used to rear flies.

### RNA extraction

Total RNA was extracted from 20-30 male and female flies using TRI reagent^® ^and the DNA-free™ kit (Ambion). Only freshly collected flies were used for RNA extraction, tissue was ground in TRI reagent^®^, and proteins and lipids were removed using a chloroform wash. Total RNA was then precipitated and cleaned with isopropanol and ethanol, respectively. Co-precipitated DNA was digested with DNase I, and the remaining RNA was again precipitated and cleaned.

### RT-PCR of ORF of *Bru-3 *mRNA

*D. pseudoobscura, D. persimilis, D. melanogaster *and *D. virilis *cDNA pools were prepared from RNA extractions using the SuperScript™ III first-strand Synthesis System for RT-PCR (Invitrogen). Reverse transcription was performed with Oligo(dT)_20 _at 50° for 50 min following the manufacturer's protocol. To amplify the putatively translated sequence of *Bru-3*, we designed primers specific to the 5' and 3'-ends of the ORF (i.e., Bru3Dpse(2)20F and Bru3-3UTR, Additional file 9), which are highly conserved among the twelve *Drosophila *species [37]. PCR was conducted in a 30 μl volume with 4 μl PCR buffer, 6 μl Q solution (Qiagen), 4.0-4.5 mM total MgCl_2_, 0.5 μM of each primer, and 3-4 units *Taq *DNA polymerase (New England BioLabs). A touchdown temperature profile was used for PCR: 10 cycles at an annealing temperature of 61° for 40 sec, 10 cycles at 60.5° for 37 sec, and finally 20 cycles at 60° for 35 sec. The denaturing and elongation temperatures were the same across all cycles: 94° for 40 sec and 72° for 2 min, respectively. 15 μl of PCR reaction was used to visualize an amplicon on a 1.5% TBE agarose gel. The remaining PCR reaction was used for cloning. We used the StrataClone™ PCR cloning vector pSC-A (Stratagene) following the manufacturer's protocol. Inserts were amplified from the transformed colonies via PCR in 30 μl, and PCR halves were arrayed on an agarose gel. The amplicons of different sizes from the same cDNA pool were preferentially sequenced to assess the diversity of ASTs expressed in adult flies. The remaining 15 μl of a PCR reaction were cleaned by applying 1.8 μl of a mixture (i.e., 1 U to 4 U) of Exonuclease I (Exo I) and Shrimp Alkaline Phosphatase (SAP, Fermentas) and incubating at 37° for 35 min. The cleaned amplicons were sequenced in both directions with BigDye v3.0 (Applied Biosystems) and run on an Applied Biosystem 3700 automated DNA sequencer.

### Alignment of ASTs of *Bru-3*

Independent clones of *Bru-3 *transcripts amplified from the same cDNA pool of *D. pseudoobscura *had different lengths, and thus were ASTs of the same gene. To identify a number of exons and positions of exon junctions in *D. pseudoobscura *mRNA, a few longest sequences of *Bru-3 *were BLASTed [BLASTN, [90]] against the genomic sequence of *D. pseudoobscura *[r2.0.1, [91]] using Drosophila Species Genomes BLAST [DroSpeGe, [92]92]. We verified that the 5' (donor) and 3' (acceptor) SSs flanking introns [i.e., GT and AG, [38]] were directly adjacent to each inferred exon junction in the genomic sequence. Then, *D. pseudoobscura *sequences were manually aligned in Se-Al v2.0 a11 [93] following the inferred positions of exon junctions in *Bru-3 *mRNA. Sequenced transcripts of *D. persimilis, D. melanogaster *and *D. virilis*, as well as two *D. melanogaster *transcripts available at FlyBase.org were added manually to the entire alignment of *D. pseudoobscura *transcripts.

### Inference of *Bru-3 *ORF from genomic sequence assemblies of *Drosophila *species

To infer the translated sequences of *Bru-3 *in twelve sequenced *Drosophila *species [37], the sequences of individual exons of *D. pseudoobscura Bru-3 *were BLASTNed [90] against the genomic sequence assemblies of *Drosophila *species (*D. melanogaster *r4.3.0, *D. simulans*, *D. sechellia*, *D. yakuba*, *D. erecta*, *D. ananassae*, *D. pseudoobscura *r2.0.1, *D. persimilis*, *D. willistoni*, *D. mojavensis*, *D. virilis*, and *D. grimshawi*) at DroSpeGe . Initially, a high statistical significance threshold [i.e., 0.01 matches were expected to be found by chance according to the model of [94]] was used for BLASTNing. If no matching sequence was found in the first round, a lower significance threshold was used for the next search. We verified that each exon junction in mRNA had the 5' and 3' SS directly adjacent to it in the genomic sequences. The exonic sequences inferred for each *Drosophila *species were concatenated and manually aligned to our *D. pseudoobscura *sequence of *Bru-3 *using Se-Al v2.0a11 [93].

### Exon turnover in *Bru-3*

Two putatively novel exons of *Bru-3*, exons 6 and 8 (Figure 1A), were identified in *D. pseudoobscura *and *D. persimilis*. These exons were absent in the two transcripts of *Bru-3 *described in *D. melanogaster *(Flybase.org). We failed to find homologous exons in the *melanogaster *group species using both BLASTNing and TBLASTNing. To confirm that the identified exons were absent from a genomic sequence and their disappearance is not a genome assembly mistake, we amplified and sequenced the homologous regions encompassing exons 6 and 8 in *D. melanogaster, D. simulans, D. erecta, D. ananassae, D. pseudoobscura, D. persimilis, D. mojavensis, D. willistoni *and *D. virilis *using primers to the conserved sequences flanking these homologous regions (Additional file 9). The PCR and sequencing of amplicons were conducted as above. The obtained sequences were aligned with the corresponding genomic regions downloaded from DroSpeGe using the web-based T-Coffee [95] under default settings. The preliminary alignments were manually edited in Se-Al.

The above assay tested for mistakes in genome assemblies only at the homologous positions. However, exons 6 and 8 could be translocated into non-homologous locations and still participate in the formation of *Bru-3 *mRNA [e.g., via trans-splicing [96]]. Therefore, we also screened the cDNA pools of *D. melanogaster, D. erecta, D. ananassae, D. mojavensis, D. willistoni*, *D. virilis *and, as a positive control, *D. pseudoobscura *for the presence of sequences similar to exons 6 and 8 using PCR. This screen assumes that the sequences of exons 6 and 8 in the tested species remained conserved to those in *D. pseudoobscura*. The primers bound to exons 6 and 9 were used to screen cDNA for exon 6. For exon 8, we used two pairs of primers: the 1^st ^pair was specific for exons 7 and 8; and the 2^nd ^bound to exons 8 and 10 (Additional file 9). PCR was performed as above, but Q solution (Qiagen) was excluded from a PCR cocktail. Amplicons were sequenced in both directions to verify their specificity. Finally, to thoroughly analyze the origin of exon 8 within the *obscura *group, both tests were also performed for the species representing its four major subgroups: the *obscura, subobscura, pseudoobscura *and *affinis *subgroups [39,40].

### Screening cDNA pools for specific exon combination

Only transcript 6, which was identified *in D. pseudoobscura*, had exons 3, 4, and 5 spliced contiguously (Table 1). We failed to sample the ASTs with this particular exon combination from any other tested species. However, one of the two known transcripts of *D. melanogaster *has this exon combination (transcript 2 in Table 1). To test for the presence of this exon combination in other *Drosophila *species, we screened cDNA pools using PCR with primers bound to exons 3 and 5 (Additional file 9). We also screened for one potential exon combination - contiguously spliced exons 5, 6, and 7 - that has not been found in any tested species (Table 1). Amplicons were checked on a 2% TBA agarose gel, purified using Exo I and SAP (Fermentas) digestion and sequenced in both directions to verify their specificity.

### Splicing constrains and molecular evolution

Splicing constraints (i.e., alternative or constitutive splicing) can affect the evolutionary potential of a gene region. Constitutively spliced regions are expected to experience stronger functional constraints than alternatively spliced regions of the same gene [e.g., [43]]. To test this prediction, we compared substitution patterns between two RRMs in *Bru-3*. Although, two regions of *Bru-3 *are homologous to the same RRM domain, and thus share the common ancestry, they are likely under different selection constrains. The C-terminal RRM of *Bru-3 *was constitutively spliced, whereas the N-terminal RRM was alternatively spliced in mRNA (Table 1). We used a conserved domain database [i.e., CDD at NCBI web page, [97]] to precisely localize two RRMs in *Bru-3 *and analyzed substitution patterns separately within both domains. The number of sites with substitutions, total number of substitutions, nucleotide diversity (¶) and its standard deviation (sd¶) were estimated in both domains among twelve *Drosophila *species [37] using DnaSP 4.20.2 [98]. In this context, we use ¶ as a measure of sequence diversity among the sampled species rather than within a species. We also calculated the ratios of synonymous/non-synonymous substitutions over the total number of synonymous/non-synonymous sites (K_S _and K_A_) for each exon between *D. pseudoobscura *and *D. melanogaster*.

### 5'- and 3'-end UTRs of *Bru-3 *in *D. pseudoobscura *and *D. persimilis*

To identify the transcribed but untranslated sequence of *Bru-3*, we used the FirstChoice^® ^RNA ligase mediated-rapid amplification of cDNA ends (RLM-RACE) kit (Ambion) following the manufacturer's directions. All RACE reactions were performed on the total RNA extracted from adult females of the *D. pseudoobscura *Flagstaff 1993 strain. Bru-3Dmel-58F and PsBru3Pout are gene-specific primers used for 5'-end and 3'-end RACE, respectively (Additional file 9). The obtained sequence allowed us to design primers specific to both untranslated regions. Bru3Dmel(UTR)+760F and Bru3Dros(1)55R were used to amplify the 5' UTR in both lines of *D. pseudoobscura*, *D. persimilis *and *D. melanogaster*. To amplify the 3' UTR from both lines of *D. pseudoobscura *and *D. persimilis*, we used PCR with Bru3end-1F and Bru3UTR-2R primers (Additional file 9). PCR was conducted as above with Q solution (Qiagen). All amplicons were digested with Exo I and SAP (Fermentas) and sequenced directly, without intermediate cloning.

## Authors' contributions

NPK conceived the study, performed the bench-work and analysis, and wrote the manuscript. MAFN supervised the analysis, wrote the script for intron analysis, and contributed to writing the manuscript. Both authors approved the final manuscript.

## Additional files

DNA sequences are deposited in GenBank [GenBank: GQ497346-GQ497445].

## Supplementary Material

Additional file 1 **The genomic position of each exon of *Bru-3 *in the twelve sequenced *Drosophila *species **[37]**at *Drosophila *Species Genomes BLAST [DroSpeGe, **[92]**]**. The table lists positions of *Bru-3 *exons in genomes of the twelve sequenced *Drosophila *species. Click here for file

Additional file 2 **Diversity of *Bru-3 *ASTs identified in each of four *Drosophila *species**. The table lists ASTs identified in each of four tested *Drosophila *species. Click here for file

Additional file 3 **cDNA screens for Bru-3 transcripts containing exon 6**. The RT-PCR gel image shows that exons 5 and 6, and exons 5, 6 and 7 are included consecutively in mature mRNA of *Bru-3 *in both lines of *D. pseudoobscura *and *D. persimilis*. Click here for file

Additional file 4 **The alignment of the genomic sequences homologous by position to exon 6 in *D. pseudoobscura *and *D. persimilis***. The color-coded alignment of genomic sequences shows the local deletions/insertions and/or substitutions at the 5' SS flaking exon 6 likely led to the loss of coding potential and subsequent decay of exon 6 in the *melanogaster *lineage: *D. anannassae, D. erecta*, *D. melanogaster *and *D. simulans*. Click here for file

Additional file 5 **The alignment of the genomic sequences homologous by position to exon 8 in *D. pseudoobscura *and *D. persimilis***. The color-coded alignment of genomic sequences shows that, while all *Drosophila *species shared both 3' and 5' SS flanking exon 8, the region corresponding to exon 8 did not encode a polypeptide chain in any tested species except *D. pseudoobscura *and *D. persimilis*. Click here for file

Additional file 6 **The alignment of the genomic sequences encompassing exon 8 from species of the *obscura *group**. The color-coded alignment of genomic sequences shows that exon 8 is conserved at the sequence level and can be translated in all tested species of the *obscura *group. Click here for file

Additional file 7 **The alignment of intron 9 for twelve *Drosophila *species **[37]. The color-coded alignment of genomic sequences shows that the species of the *melanogaster *group, *D. simulans, D. sechelia, D. erecta, D. yakuba, D. melanogaster *and *D. ananassae*, evolved an alternative 3' SS of intron 9. Click here for file

Additional file 8 **Intron sizes of *Bru-3 *in the twelve sequenced *Drosophila *species **[37]**at *Drosophila *Species Genomes BLAST [DroSpeGe**, [92]**]**. The table lists *Bru-3 *intron lengths for the twelve sequenced *Drosophila *species. Click here for file

Additional file 9 **Primer applications and sequences**. The table lists the sequences of primers used in this study. Click here for file
