# Supplementary material for: Large introns in relation to alternative splicing and gene evolution: a case study of Drosophila bruno-3
Source: BMC Genet. 2009 Oct 19;10:67. doi: 10.1186/1471-2156-10-67 (PMC2767349; doi:10.1186/1471-2156-10-67)
Supplement: Additional file 2 — Diversity of Bru-3 ASTs identified in each of four Drosophila species. The table lists ASTs identified in each of four tested Drosophila species. [file 1471-2156-10-67-S2.PDF]

Additional file 2 — Diversity of *Bru-3* ASTs identified in each of four *Drosophila* species

		Exons in ORF														
AST	Size (bp)	2	3	4	4a	5	6	7	8	9	10	11	12	13	14	Frequency
	D. pseudoobscura															
1	1281	60	–	49	–	135	–	80	57	144	274	99	78	177	128	1
2	1224	60	–	49	–	135	–	80	–	144	274	99	78	177	128	4
3	1206	60	–	49	–	135	–	80	–	144	274	99	78	159	128	3
4	1188	60	42	49	–	135	–	80	–	144	274	99	–	177	128	1
5	1146	60	–	49	–	135	–	80	–	144	274	99	–	177	128	8
6	1146+454	60	–	49	454	135	–	80	–	144	274	99	–	177	128	3
7	1131	60	42	49	–	–	–	80	–	144	274	99	78	177	128	1
8	1128	60	–	49	–	135	–	80	–	144	274	99	–	159	128	1
9	1122	60	–	49	–	–	51	80	–	144	274	99	78	159	128	1
10	1122	60	–	31	–	–	51	80	–	144	274	99	78	177	128	1
11	1113	60	42	49	–	–	–	80	–	144	274	99	78	159	128	1
12	1089	60	–	49	–	–	–	80	–	144	274	99	78	177	128	8
13	1071	60	–	49	–	–	–	80	–	144	274	99	78	159	128	3
14	1071	60	–	31	–	–	–	80	–	144	274	99	78	177	128	1
15	1062	60	–	49	–	–	51	80	–	144	274	99	–	177	128	1
16	1062+454	60	–	49	454	–	51	80	–	144	274	99	–	177	128	1
17	1059	60	–	49	–	–	–	80	–	144	274	69	78	177	128	1
18	1044	60	–	49	–	–	51	80	–	144	274	99	–	159	128	1
19	1026	60	–	31	–	–	51	80	–	144	274	99	–	159	128	1
20	1011	60	–	49	–	–	–	80	–	144	274	99	–	177	128	7
21	1011+452	60	–	49	452	–	–	80	–	144	274	99	–	177	128	1
22	993	60	–	49	–	–	–	80	–	144	274	99	–	159	128	1
23	?					135	51	80								X
	D. persimilis															
1	1224	60	–	49	–	135	–	80	–	144	274	99	78	177	128	2
5	1146	60	–	49	–	135	–	80	–	144	274	99	–	177	128	1
2	1104	60	42	49	–	–	–	80	–	144	274	99	–	177	128	1
3	1089	60	–	49	–	–	–	80	–	144	274	99	78	177	128	4
4	1089+452	60	–	49	452	–	–	80	–	144	274	99	78	177	128	1
5	1011	60	–	49	–	–	–	80	–	144	274	99	–	177	128	1
6	414	60	–	49	–	–	–	–	–	–	–	–	–	177	128	1
7	?		42	49	–	135										X
8	?						51	80	–	144						X
9	?							80	57	144	274					X
10	?					135	51	80								X
	D. melanogaster															
1, A	1269	60	42	49	–	135	N/A	80	N/A	144	277	99	78	177	128	0
2	1251	60	–	49	–	135	N/A	80	N/A	144	301	99	78	177	128	1
3	1227	60	–	49	–	135	N/A	80	N/A	144	277	99	78	177	128	2
4	1173	60	–	49	–	135	N/A	80	N/A	144	301	99	–	177	128	1
5	1155	60	–	31	–	135	N/A	80	N/A	144	301	99	–	177	128	1
6	1149	60	–	49	–	135	N/A	80	N/A	144	277	99	–	177	128	3

7, B	1092	60	–	49	–	–	N/A	80	N/A	144	277	99	78	177	128	1
8	1038	60	–	49	–	–	N/A	80	N/A	144	301	99	–	177	128	1
9	1014	60	–	49	–	–	N/A	80	N/A	144	277	99	–	177	128	2
10	1014+454	60	–	49	454	–	N/A	80	N/A	144	277	99	–	177	128	1
11	996	60	–	49	–	–	N/A	80	N/A	144	277	99	–	159	128	1
12	831	60	–	–	–	–	N/A	80	N/A	144	277	99	–	177	128	1
13	378+454	60	–	31	454	–	N/A	–	N/A	–	–	–	–	159	128	1
14	549	60	–	49	–	135	N/A	–	N/A	–	–	–	–	177	128	1
15	414	60	–	49	–	–	N/A	–	N/A	–	–	–	–	177	128	2
	?		42	49	–	135			N/A							X
	D. virilis															
1	1224	60	–	49	–	135	–	80	N/A	144	274	99	78	177	128	4
2	1206	60	–	49	–	135	–	80	N/A	144	274	99	78	159	128	3
3	1146	60	–	49	–	135	–	80	N/A	144	274	99	–	177	128	2
4	1089	60	–	49	–	–	–	80	N/A	144	274	99	78	177	128	2
5	1071	60	–	49	–	–	–	80	N/A	144	274	99	78	159	128	1
6	1053	60	42	49	–	–	–	80	N/A	144	274	99	–	177	128	1
7	1011	60	–	49	–	–	–	80	N/A	144	274	99	–	177	128	5
8	993	60	–	49	–	–	–	80	N/A	144	274	99	–	159	128	1
9	?						51	80	N/A	144						X
10	?		42	49	–	135			N/A							X

Alternatively spliced transcripts (AST) were sampled by sequencing multiple clones of the entire ORF of *Bru-3* for four different species of *Drosophila*: *D. pseudoobscura*, *D. persimilis, D. melanogaster* and *D. virilis.* Two known ASTs of *D. melanogaster* (FlyBase.org) labeled with letters – A and B – are also includes in the table. The found ASTs and their frequencies are presented individually for each *Drosophila* species. Sizes of each distinct AST and exon are presented in nucleotide numbers. The long dash designates an exon-skipping event. A few unique combination of exons designated by “X” were found in cDNA pools using specific primers (see test for details).
