# Supplementary material for: Large introns in relation to alternative splicing and gene evolution: a case study of Drosophila bruno-3
Source: BMC Genet. 2009 Oct 19;10:67. doi: 10.1186/1471-2156-10-67 (PMC2767349; doi:10.1186/1471-2156-10-67)
Supplement: Additional file 3 — cDNA screens for Bru-3 transcripts containing exon 6. The RT-PCR gel image shows that exons 5 and 6, and exons 5, 6 and 7 are included consecutively in mature mRNA of Bru-3 in both lines of D. pseudoobscura and D. persimilis. [file 1471-2156-10-67-S3.PDF]

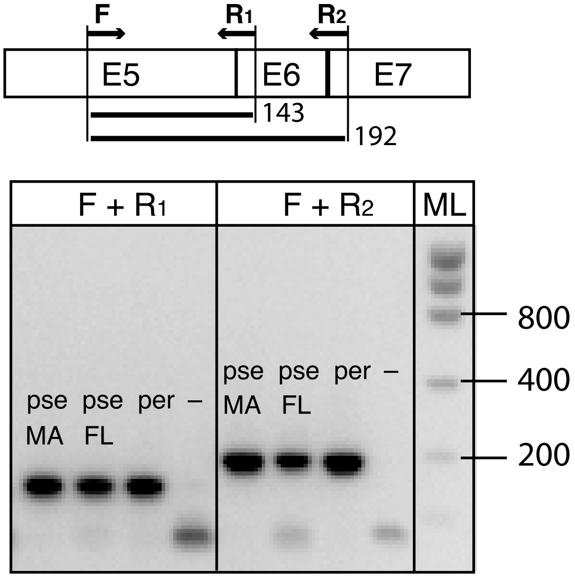

Additional file 3 — cDNA screens for *Bru-3* transcripts containing exon 6. In the diagrams, arrows indicate the position and direction of primers in regard to exons (e.g. E5, E6, and E7) used for PCR. The sequences of the primers used in the screens are listed in supplementary table S1. The expected sizes of PCR products (in nucleotides) are presented below the diagram. In the gel images, fly lines are indicated for each track. The long dash marks the track of a negative control (no template). Sizes of DNA ladder (ML) are shown to the right of the gel image. Transcripts containing exons 5 and 6, as well as exons 5, 6 and 7 were found in cDNA pools from both lines of *D. pseudoobscura* (pse) – Mather 17 and Flagstaff 1993 (MA and FL) lines – and *D. persimilis* (per).
