# Supplementary material for: Large introns in relation to alternative splicing and gene evolution: a case study of Drosophila bruno-3
Source: BMC Genet. 2009 Oct 19;10:67. doi: 10.1186/1471-2156-10-67 (PMC2767349; doi:10.1186/1471-2156-10-67)
Supplement: Additional file 4 — The alignment of the genomic sequences homologous by position to exon 6 in D. pseudoobscura and D. persimilis. The color-coded alignment of genomic sequences shows the local deletions/insertions and/or substitutions at the 5' SS flaking exon 6 likely led to the loss of coding potential and subsequent decay of exon 6 in the melanogaster lineage: D. anannassae, D. erecta, D. melanogaster and D. simulans. [file 1471-2156-10-67-S4.PDF]

**Additional file 4 — The alignment of the genomic sequences homologous by position to exon 6 in *D. pseudoobscura* and *D. persimilis*.** Only the focal part of this alignment immediately at and near exon 6 is presented. Genomic fragments were amplified and sequenced from the corresponding *Drosophila* species, except for the sequences of *D. ananassae* and *D. willistoni*, which were downloaded from FlyBase.org (see test for details). Exon 6 is highlighted in grey. The splicing sites around exon 6 are underscored. We found that exon 6 was transcribed in *D. pseudoobscura, D. persimilis, D. mojavensis,* and *D. virilis* adult flies (Fig. 2A, B). While this exon was conserved in *D. willistoni*, the local deletions/ insertions and/or substitutions at the 5’ SS flaking exon 6 likely led to the loss of coding potential and subsequent decay of exon 6 in the *melanogaster* lineage: *D. anannassae, D. erecta*, *D. melanogaster* and *D. simulans*.

```
D. simulans         1 TCACCCTTCCTAGGATTTATCTTATTTTCTGTTTTAGA----------------------
D. melanogaster     1 TCACACTGTCTAGGATTTATCGTATTTTCTGTTTTAGA----------------------
D. erecta           1 TCCCACACCCTGCGATTTTTCCTATACTTTGTTTTAGG----------------------
D. ananassae        1 CAAAGCCCTTGGCGAATTATACTTTTCATTGTTTTAGGATCGAAAGAGGAGCCCAAAGAG
D. pseudoobscura    1 CGAACTCATTGAGTTTTCATTCTTCTCTCTGTTTTAGG----------------------
D. persimilis       1 CGAACTCATTGAGTTTTCATTCTTCTCTCTGTTTTAGG----------------------
D. willistoni       1 CTAATTCTCTTTTTCTTCTCTTTTTACCATTTTTTAGG----------------------
D. mojavensis       1 TATCGTATTAATTTATTGAATTCTCTTCTCTTTTTAGG----------------------
D. virilis          1 TTTACTAATAATTTTATGCACTTTCTTATCTTTTTAGG----------------------

D. simulans        39 -----------------------------------TCAGTTCGCCAGGTTTTAT------
D. melanogaster    39 -----------------------------------GAAGTTTGCCACGTTTTAT------
D. erecta          39 -----------------------------------TAAGTGCGTCTGGTTTTC-------
D. ananassae       61 TACATCTGCCGCTTGTTCATAAACCATATATCAGCAAAGCTCATTAGTTTTATTCTGCC
D. pseudoobscura   39 -----------------------------------TAAGTGCACAAGGCTTCAT------
D. persimilis      39 -----------------------------------TAAGTGCACAAGGCTTCAT------
D. willistoni      39 -----------------------------------TAAGTGCACAAGGCTTCAT------
D. mojavensis      39 -----------------------------------TAAGTGCACTAGGCTTCAT------
D. virilis         39 -----------------------------------TAAGTGCACAAGGCTTCAT------

D. simulans        58 ----------------------------------CCTTACAGCTGAGCCC-AAATTCACT
D. melanogaster    58 ----------------------------------CCTTACAGCAGAGCCAAAAATTCAAT
D. erecta          57 ----------------------------------CCTTGCAGCTTAACCCGAAATTCACT
D. ananassae      121 CTTGTGACTGTAATTGGCCGAGAATGGAGAACAAGCTAGGAACATAATTCCTCATTCAAT
D. pseudoobscura   58 ----------------------------------CCTTACAACAGAACACGTGATCCACG
D. persimilis      58 ----------------------------------CCTTACAACAGAACACGTGATCCACG
D. willistoni      58 ----------------------------------CCTTACACCAGAACACGTGATCCACG
D. mojavensis      58 ----------------------------------CCTTACAACAAGACACGTGCTCCACG
D. virilis         58 ----------------------------------CCTTACAACAAAACACGTCATACACG

D. simulans        83 CAAAGAATTTATCTAAGAAATTAAAATT
D. melanogaster    84 TAACGAATATTTCTAAGAAATTAAAATT
D. erecta          83 CAAAGAATATTTCTAAGATGTTAAAATT
D. ananassae      181 TGAAACTTCAAGTTTGGAATTCCAAGCT
D. pseudoobscura   84 TGGCGGTAAGCAGATGA-CAAACACTAT
D. persimilis      84 TGGCGGTAAGCAGATGA-CAAACACTAT
D. willistoni      84 TGGCGGTAAGCAGTTGATCTAACAATAT
D. mojavensis      84 TGGCGGTAAGCAGTTGT-CTTACAATAT
D. virilis         84 TGGCGGTAAGCAGCTGC-CTTGAAATAT
```
