# Supplementary material for: Large introns in relation to alternative splicing and gene evolution: a case study of Drosophila bruno-3
Source: BMC Genet. 2009 Oct 19;10:67. doi: 10.1186/1471-2156-10-67 (PMC2767349; doi:10.1186/1471-2156-10-67)
Supplement: Additional file 5 — The alignment of the genomic sequences homologous by position to exon 8 in D. pseudoobscura and D. persimilis. The color-coded alignment of genomic sequences shows that, while all Drosophila species shared both 3' and 5' SS flanking exon 8, the region corresponding to exon 8 did not encode a polypeptide chain in any tested species except D. pseudoobscura and D. persimilis. [file 1471-2156-10-67-S5.PDF]

Additional file 5 — The alignment of the genomic sequences homologous by position to exon 8 in *D. pseudoobscura* and *D. persimilis*. Only the focal part of this alignment immediately at and near exon 8 is presented. Genomic fragments were amplified and sequenced from the corresponding *Drosophila* species, except for the sequence of *D. willistoni*, which were downloaded from FlyBase.org (see test for details). Exon 8 is highlighted in grey. The splicing sites around exon 8 are underscored. While all *Drosophila* species shared both 3' and 5' SS flanking exon 8, the region corresponding to exon 8 did not encode a polypeptide chain in any tested species except *D. pseudoobscura* and *D. persimilis.* Notably, sequence changes that capacitated exon 8 in the *obscura* group were highly focal and could not be attributed to duplication-translocation of existing exons [e.g., 1, 2], retrotransposition [e.g., 3] or exaptation of repetitive elements [e.g., *Alu* element, 4, 5]. Therefore, the functional ORF of exon 8 was created *de novo* from intronic sequence between the existing SS.

```
D. simulans         1 AATCATTATACCCTTTTCT-TTTGTCGTTACAGGTATGTAAAAA---------------A
D. melanogaster     1 AATCATCATACCCTTTTCT-TTTGTCGTTACAGGTATGTAAAAAGAA------------A
D. erecta           1 AATCATAATACCCTTTTCTCTTTGTCTTTACAGGTATGTACAA-----------------
D. ananassae        1 TCTAAATGTATTCTTATCCTTCTCTCTTTTCAGGTATGTTGAA----------------A
D. pseudoobscura    1 TAAATCTGATTTTCCCTCTCTCTATCTTTGCAGGTATGTACTA---------------CA
D. persimilis       1 TAAATCTGATTTTCCCTCTCTCTATCTTTGCAGGTATGTACTA---------------CA
D. willistoni       1 TAACCTCTATTTTCTTTTCTCTTATCGTTTCAGGTATGTAACAAAAAAAAAGTGATGGGA
D. mojavensis       1 TATCACTTCTCTTCTTCTTCTTTATCGTTGCAGGTATGTACCAACAACAA--------CA
D. virilis          1 TCATATTTATTCTCTTCTTCTTTATCTTTACAGGTATGTACTA---------------CA


D. simulans        45 AA-GGTCTTAACCCTCGAGATAAT-CAATCCT----------------------------
D. melanogaster    48 AA-GGTCTAAACCCTCGAGATAAT-CAATCCT----------------------------
D. erecta          44 AA-GGTCCTAACCCCCAGGATAAT-CAATCCT----------------------------
D. ananassae       45 AATTAGCATAACACAAT-AATAA-CCAATCGG----------------------------
D. pseudoobscura   46 AATTTGCATAGCTCCAAGGAT-AT-CAATCCTTT--GCCCTATT----------------
D. persimilis      46 AATTTGCATAGCTCCAAGGAT-AT-CAATCCTTT--GCCCTATT----------------
D. willistoni      61 AATTTGCATAAATCTCTCTCGAATCCAACCCTTTTTTTCCTATTCCCTACTATCCCACAC
D. mojavensis      53 AATTTGCATAACACAATGCAT----GAAAAAAATAGAAAAAAAGTAATCGA--GTAAAAA
D. virilis         46 AATTTGCATAACACGA-------------------------------------GCAAAGA


D. simulans        75 ------------TAAAGTGTTCC-----GTAAGTTCAAGTGCCTTTGTCTTGTCC
D. melanogaster    78 ------------TAAAGTGTTCC-----GTAAGTTCAAGTGCCTTTGTCTTGTGC
D. erecta          74 ------------TAAAGTGTTCC-----GTAAGTTCAAGTGCCTTTGTCTTGTCC
D. ananassae       75 ------------TCAAGTTTTCC-----GTAAGTTCAAGTGCCGTTGTCTGGCGG
D. pseudoobscura   86 ------------------CTGGC-----GTAAGTTCAAGTGCCTTTATCTTGTCC
D. persimilis      86 ------------------CTGGC-----GTAAGTTCAAGTGCCTTTATCTTGTCC
D. willistoni     121 ACTATATATCCAACAAATGCTCC---AAGTAAGTTCAAGTGTCTCTTCTATTCTG
D. mojavensis     107 AGAACC-CTTTCGAGGGGCATCCTTTATGTAAGTGCAGTGTCGCAGACAAATCCT
D. virilis         69 ATAACCCCTTT---GGGGCATCCTTTATGTAAGTGCAGTGTCCCAGACGAATCCT
```

References

1. Nurminsky DI, Nurminskaya MV, Aguiar DD, Hartl DL: **Selective sweep of a newly evolved sperm-specific gene in *Drosophila***. *Nature* 1998, **396**(6711):572-575.
2. Chen S-T, Cheng H-C, Barbash DA, Yang H-P: **Evolution of *hydra*, a recently evolved testis-expressed gene with nine alternative first exons in *Drosophila melanogaster***. *PLoS Genetics* 2007, **3**(7):e107.

3. Betran E, Wang W, Jin L, Long M: **Evolution of the *Phosphoglycerate mutase* processed gene in human and chimpanzee revealing the origin of a new primate gene**. *Mol Biol Evol* 2002, **19**(5):654-663.
4. Lev-Maor G, Sorek R, Shomron N, Ast G: **The birth of an alternatively spliced exon: 3' splice-site selection in Alu exons**. *Science* 2003, **300**(5623):1288-1291.
5. Sorek R, Lev-Maor G, Reznik M, Dagan T, Belinky F, Graur D, Ast G: **Minimal conditions for exonization of intronic sequences: 5' splice site formation in *Alu* exons**. *Mol Cell* 2004, **14**(2):221-231.
