# Supplementary material for: Large introns in relation to alternative splicing and gene evolution: a case study of Drosophila bruno-3
Source: BMC Genet. 2009 Oct 19;10:67. doi: 10.1186/1471-2156-10-67 (PMC2767349; doi:10.1186/1471-2156-10-67)
Supplement: Additional file 6 — The alignment of the genomic sequences encompassing exon 8 from species of the obscura group. The color-coded alignment of genomic sequences shows that exon 8 is conserved at the sequence level and can be translated in all tested species of the obscura group. [file 1471-2156-10-67-S6.PDF]

Additional file 6 — The alignment of the genomic sequences encompassing exon 8 from species of the *obscura* group. Only the focal part of this alignment immediately at and near exon 8 is presented. All genomic fragments were amplified and sequenced from species of the *obscura* group. Exon 8 is highlighted in grey. The splicing sites around exon 8 are underscored. Each tested species had a valid ORF in exon 8. In addition, we found that exon 8 was transcribed in *D. pseudoobscura* and *D. persimilis*, *D. obscura, D. bifasciata, D. subobscura* and *D. affinis* adult flies (fig. 2B). Because these species represent all major branches within the *obscura* group [1, 2], exon 8 originated very early in the lineage of the *obscura* group. *D. pseudoobcura bogotana* Ayala & Dobzhansky, 1974 (bog.) is a geographically isolated subspecies of *D. pseudoobcura.*

```
D. pseudoobscura       1 ATTTTCCCTCTCTC----------TATCTTTGCAGGTATGTACTACAAATTTGCATAGCT
D. pseudoobscura bog.  1 ATTTTCCCTCTCTC----------TATCTTTGCAGGTATGTACTACAAATTTGCATAGCT
D. persimilis          1 ATTTTCCCTCTCTC----------TATCTTTGCAGGTATGTACTACAAATTTGCATAGCT
D. miranda             1 ATTTTCCCTCTCTC----------TATCTTTGCAGGTATGTACTACAAATTTGCATAGCT
D. obscura             1 ATTTTCC-TCTCTCTCTTTCTATATATATTTTCAGGTATGTACTACAAATTTGCATAGCT
D. subobscura          1 ATTTTCC-TCTCTCTCTTTCTATATATATTTTCAGGTATGTACTACAAATTTGCATAGCT
D. bafasciata          1 ATTTTGCCTCTCTC----------TATCTTTGCAGGTATGTACCACAAATTTGCATAGCT
D. affinis             1 ATTTTCCCTCT---TCTTTCTC--TGTCGTTGCAGGTATGTACTACAAATTTGCATAGCT

D. pseudoobscura      51 CCAAGGATATCAATCCTTTGCCCTATTCTGGCGTAAGTTCAAGTGCCTTTATCTTGTCCC
D. pseudoobscura bog.51 CCAAGGATATCAATCCTTTGCCCTATTCTGGCGTAAGTTCAAGTGCCTTTATCTTGTCCC
D. persimilis         51 CCAAGGATATCAATCCTTTGCCCTATTCTGGCGTAAGTTCAAGTGCCTTTATCTTGTCCC
D. miranda            51 CCAAGGATATCAATCCTTTGCCCTATTCTGGCGTAAGTTCAAGTGCCTTTATCTTGTCCC
D. obscura            60 CCAAGGATATCAATCCTTTGCCCTATTCGAGCGTAAGTTCAAGTGCCTTTGTCTAGCCCC
D. subobscura         60 CCAAGGATATCAATCCTTTGCCCTATTCGAGCGTAAGTTCAAGTGCCTTTGTCTAGCCCC
D. bafasciata         51 CCAAGGATATCAATCCTTTGCCCTATTCTGTCGTAAGTTCAAGTGCCTTTGTCTTGTCCC
D. affinis            56 CCAAGGATATCAATCCTTTGCCCTGTTCTGGCGTAAGTTCAAGTGCCTTTATCTTGTCCC
```


References

1. O'Grady PM: **Reevaluation of phylogeny in the *Drosophila obscura* species group based on combined analysis of nucleotide sequences**. *Mol Phylog Evol* 1999, **12**(2):124-139.

2. Prud'homme B, Gompel N, Rokas A, Kassner VA, Williams TM, Yeh S-D, True JR, Carroll SB: **Repeated morphological evolution through cis-regulatory changes in a pleiotropic gene**. *Nature* 2006, **440**(7087):1050–1053.
