# Supplementary material for: Large introns in relation to alternative splicing and gene evolution: a case study of Drosophila bruno-3
Source: BMC Genet. 2009 Oct 19;10:67. doi: 10.1186/1471-2156-10-67 (PMC2767349; doi:10.1186/1471-2156-10-67)
Supplement: Additional file 7 — The alignment of intron 9 for twelve Drosophila species [37]. The color-coded alignment of genomic sequences shows that the species of the melanogaster group, D. simulans, D. sechelia, D. erecta, D. yakuba, D. melanogaster and D. ananassae, evolved an alternative 3' SS of intron 9. [file 1471-2156-10-67-S7.PDF]

Additional file 7 — The alignment of intron 9 for twelve *Drosophila* species [1]. The entire sequences of intron 9 were downloaded from FlyBase.org. All twelve *Drosophila* species share the conserved 5' and 3' splice sites (SSs) of intron 9, the pairs of underscored nucleotides that flank each sequence. The species of the *melanogaster* group, *D. simulans, D. sechelia, D. erecta, D. yakuba, D. melanogaster* and *D. ananassae,* evolved an alternative 3' SS, the nucleotide pair highlighted in grey. The splicing at the alternative 3' SS results in the longer exon 10 (see test for details).

```
D_simulans       1 GTAAGT------CCGCT-CCCCCAT-TCCCGCGGCAATCA-CCCACCTAAATATAAAGC
D_sechellia      1 GTAAGT------CCGCT-CTCCCAT-CCCCGCGGCAATCA-CCCACCTAAATATAAAGC
D_erecta         1 GTAAGT------CCGCTCCCCCCA-CCCC-AGCGCACTCA-CCCACCTATATATAAAGC
D_yakuba         1 GTAAGT------CCGCTCCCCCCATCCCCAAATGCATTCACCGCTCCTACATATAAAGC
D_melanogaster   1 GTAAGT------CCGCT-TCCCCAT-TACCGCGGCAATCA-TCCACCTAAATATAAAGC
D_ananassae      1 GTAAGT------------------CGACCACTCCCCCTCTCTCATCAAGAATGCCCCTA
D_pseudoobscura  1 GTAAGT----------------------------------GGGATGCAGCTCCAGCTCC
D_persimilis     1 GTAAGT----------------------------------GGGATGCAGCTCCAGCTCC
D_willistoni     1 GTAAGTGAAAGATAACATTTTTGCGACTCTACATAAAATCCTTGCATCACCTGCCAATT
D_mojavensis     1 GTAAGT----------------------------AGCCGTGCACCCGCACACACAGCCT
D_virilis        1 GTAAGT---------------------------------ACCCTCCTCACCCATACCCG
D_grimshawi      1 GTAAAT---------------------------------AGCCAACTCTCTTTTCACCC

D_simulans      51 AG------------CTTCCCTCTCCTCCG------GAA---TTGCAG
D_sechellia     51 AG------------CTTCCCTCTCCTCCG------GAA---TTGCAG
D_erecta        51 AG------CTCCCCCTCCCCTCTCCTCCG------GAA---TTGCAG
D_yakuba        54 AG------------CTCCCCTCTCCTCCG------GAA---TTGCAG
D_melanogaster  51 AG------------CTTCCCTCTCCTCCG------GAA---TTGCAG
D_ananassae     42 AG------------------CCTCCTCCC------------TTGCAG
D_pseudoobscura 26 AGCTCCATACCCTCTTACGCCCACCATCTTATTTCGAATCTCTACAG
D_persimilis    26 AGCTCCATCTCCTCTTACGCCCACCATCTTTTTTCGAATCTCTACAG
D_willistoni    60 TGCCAACTTGGTTTTTTTTGTTTCTCCTTTAATGTCAACCGACACAG
D_mojavensis    32 CACAGTCACTTCAGTCAGCTTGATTGCAATCCTTTGTGCCTCATTAG
D_virilis       27 CCAACGGCCTCATTACGCCCATTTGTGTTTCTCCAAAAAAAAAACAG
D_grimshawi     27 TGTTCCGCATAGTAATCATCTCATTACATTTTTTCCGTCGCTTACAG
```

References

1. Clark AG, Eisen MB, Smith DR, Bergman CM, Oliver B, Markow TA, Kaufman TC, Kellis M, Gelbart WM: **Evolution of genes and genomes on the Drosophila phylogeny**. *Nature* 2007, **450**(7167):203-218.
