# Supplementary material for: Large introns in relation to alternative splicing and gene evolution: a case study of Drosophila bruno-3
Source: BMC Genet. 2009 Oct 19;10:67. doi: 10.1186/1471-2156-10-67 (PMC2767349; doi:10.1186/1471-2156-10-67)
Supplement: Additional file 8 — Intron sizes of Bru-3 in the twelve sequenced Drosophila species [37]at Drosophila Species Genomes BLAST [DroSpeGe, [92]]. The table lists Bru-3 intron lengths for the twelve sequenced Drosophila species. [file 1471-2156-10-67-S8.PDF]

Additional file 8 — Intron sizes of *Bru-3* in the twelve sequenced *Drosophila* species [1] at *Drosophila* Species Genomes BLAST [DroSpeGe, 2].

Drosophila	Intron number													
species	1	2	3	4	5	6	7	8	9	10	11	12	13	14
D. ananassae	N/A	12621	13149	41176	17763	N/A	38184	N/A	43	334	1054	1376	61	N/A
D. erecta	N/A	13033	12884	38102	18634	N/A	36969	N/A	46	117	839	1407	73	N/A
D. grimshawi	N/A	17798	16178	44277	8472	12519	47550	N/A	73	196	1117	1590	79	N/A
D. melanogaster	1511	12822	12989	36040	18070	N/A	41973	N/A	52	134	847	1444	71	249
D. mojavensis	N/A	18992	20852	44473	8499	17651	47397	N/A	78	93	1137	1438	78	N/A
D. persimilis	1772	14076	14529	40571	8110	11218	31908	10415	72	129	1021	1657	68	181
D. pseudoobscura	1716	14086	14568	40650	8213	11323	32092	10467	72	129	1024	1565	68	181
D. sechellia	N/A	13449	12776	36076	17899	N/A	36267	N/A	52	133	855	1453	71	N/A
D. simulans	N/A	12963	14201	41532	17946	N/A	22381	N/A	52	133	848	1456	71	N/A
D. virilis	N/A	17867	15153	48738	8463	11886	45859	N/A	73	160	1181	1566	100	N/A
D. willistoni	N/A	18818	19118	49153	10160	13309	49315	N/A	106	253	1233	2502	78	N/A
D. yakuba	N/A	13330	13081	37730	18484	N/A	38494	N/A	55	106	824	1453	73	N/A

References

1. Clark AG, Eisen MB, Smith DR, Bergman CM, Oliver B, Markow TA, Kaufman TC, Kellis M, Gelbart WM: **Evolution of genes and genomes on the Drosophila phylogeny**. *Nature* 2007, **450**(7167):203-218.
2. Gilbert DG: **DroSpeGe: rapid access database for new Drosophila species genomes**. *Nucl Acids Res* 2007, **35**(suppl_1):D480-485.
