# Supplementary material for: Large introns in relation to alternative splicing and gene evolution: a case study of Drosophila bruno-3
Source: BMC Genet. 2009 Oct 19;10:67. doi: 10.1186/1471-2156-10-67 (PMC2767349; doi:10.1186/1471-2156-10-67)
Supplement: Additional file 9 — Primer applications and sequences. The table lists the sequences of primers used in this study. [file 1471-2156-10-67-S9.PDF]

Additional file 9 — Primer applications and sequences

Primer	Application	Sequence
Bru3Dpse(1)20F	entire ORF	TGGTCGGCCAGCCGAAGGTTGAGTTTGCTC
Bru3-3UTR	entire ORF	GATTGTTAATAGGGTCGACTGGCATCCTTTGGCCGCTTC
Exon6-genomF	genomic sequence around exon 6	TTATTCCACTTTTGTTGTTAGACAATATCC
Exon6-genomR	genomic sequence around exon 6	ATGTGATTTTTGATTGAGTGCCGACCAAACAG
Exon8-genomF	genomic sequence around exon 8	GGTCAAAGACAATATTGAAATGCCAATTGAAC
Exon8-genomR	genomic sequence around exon 8	AGCTTGTAATCACTGTCAATGTCGAC
Bru3Dpse(6)-287R	screen for transcription of exon 6	GTAAGTGCACAAGGCTTCATCCTTACA
Bru3Dpse(9)-592R	screen for transcription of exon 6	ACTGATTAAAGACAAACGGATTCAGCAGGT
Bru3Dpse(7)-347F	1st screen for transcription of exon 8	TCGTCAAATTCGGAACGCAACAGGAGGCTCA
Bru3Dpse(8)-466R	1st screen for transcription of exon 8	AGGGCAAAGGATTGATATCCTTGGAGCTAT
Bru3Dpse(8)-435F	2nd screen for transcription of exon 8	GCATAGCTCCAAGGATATCAATCCTTTGCCCTA
Bru3Dpse(10)807R	2nd screen for transcription of exon 8	CTGGCCGTTGGGCGTGTTGGCGCCCAT
Bru-3Dmel-58R	5’-end RACE	TAACTTGTTGAGCAAACTCAACCTTC
PsBru3POutF	3’-end RACE	GATCAGCTCCAAGGTCTTCATCGATC
Bru3Dmel(UTR)+760F	5’-end UTR	GGAATATTTCGTATAGTTCTCGTCGACTGTCGTCTGA
Bru3Dros(1)55R	5’-end UTR	CTTGTTGAGCAAACTCAACCTTCGGCTGGCCGACCA
Bru3end-1F	3’-end UTR	GAAGCGGCCAAAGGATGCCAGTCGACCCTA
Bru3UTR-2R	3’-end UTR	GCAGTTGTCATGGTTGCTGTTGTGGGTCCTGGT
Bru3Dpse(3)-68F	contiguous splicing of exons 3, 4, 5	GCCATCATCCATTAATACCACCAGCCATCA
Bru3Dros(5)-189R	contiguous splicing of exons 3, 4, 5	GCTGAGCATGCCAACGAASAGCTTGCGATCT
Bru3Dpse(5)-160F	contiguous splicing of exons 5, 6, 7	GATCGCAAGCTCTTCGTTGGCATGCTCAGCA
Bru3Dpse(6/7)-352R	contiguous splicing of exons 5, 6, 7	TGACGAATGCGCAGCCGCCACGTGGA
